# RMVar: an updated database of functional variants involved in RNA modifications

**DOI:** 10.1093/nar/gkaa811

**Published:** 2020-10-06

**Authors:** Xiaotong Luo, Huiqin Li, Jiaqi Liang, Qi Zhao, Yubin Xie, Jian Ren, Zhixiang Zuo

**Affiliations:** State Key Laboratory of Oncology in South China, Cancer Center, Collaborative Innovation Center for Cancer Medicine, School of Life Sciences, Sun Yat-sen University, Guangzhou 510060, China; State Key Laboratory of Oncology in South China, Cancer Center, Collaborative Innovation Center for Cancer Medicine, School of Life Sciences, Sun Yat-sen University, Guangzhou 510060, China; State Key Laboratory of Oncology in South China, Cancer Center, Collaborative Innovation Center for Cancer Medicine, School of Life Sciences, Sun Yat-sen University, Guangzhou 510060, China; State Key Laboratory of Oncology in South China, Cancer Center, Collaborative Innovation Center for Cancer Medicine, School of Life Sciences, Sun Yat-sen University, Guangzhou 510060, China; Precision Medicine Institute, The First Affiliated Hospital, Sun Yat-sen University, Guangzhou 510060, China; State Key Laboratory of Oncology in South China, Cancer Center, Collaborative Innovation Center for Cancer Medicine, School of Life Sciences, Sun Yat-sen University, Guangzhou 510060, China; State Key Laboratory of Oncology in South China, Cancer Center, Collaborative Innovation Center for Cancer Medicine, School of Life Sciences, Sun Yat-sen University, Guangzhou 510060, China

## Abstract

Distinguishing the few disease-related variants from a massive number of passenger variants is a major challenge. Variants affecting RNA modifications that play critical roles in many aspects of RNA metabolism have recently been linked to many human diseases, such as cancers. Evaluating the effect of genetic variants on RNA modifications will provide a new perspective for understanding the pathogenic mechanism of human diseases. Previously, we developed a database called ‘m6AVar’ to host variants associated with m^6^A, one of the most prevalent RNA modifications in eukaryotes. To host all RNA modification (RM)-associated variants, here we present an updated version of m6AVar renamed RMVar (http://rmvar.renlab.org). In this update, RMVar contains 1 678 126 RM-associated variants for 9 kinds of RNA modifications, namely m^6^A, m^6^Am, m^1^A, pseudouridine, m^5^C, m^5^U, 2′-O-Me, A-to-I and m^7^G, at three confidence levels. Moreover, RBP binding regions, miRNA targets, splicing events and circRNAs were integrated to assist investigations of the effects of RM-associated variants on posttranscriptional regulation. In addition, disease-related information was integrated from ClinVar and other genome-wide association studies (GWAS) to investigate the relationship between RM-associated variants and diseases. We expect that RMVar may boost further functional studies on genetic variants affecting RNA modifications.

## Graphical Abstract

**Graphical Abstract:**
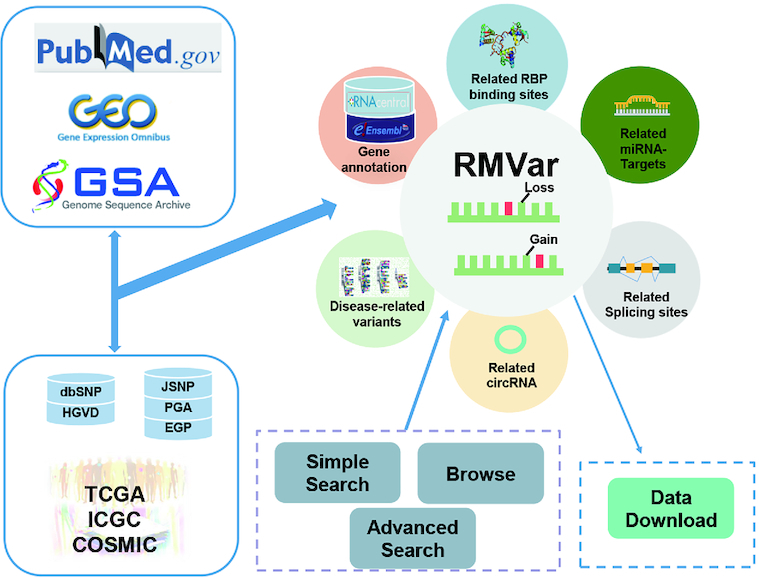
RMVar: an updated database of functional variants involved in RNA modifications.

## INTRODUCTION

With the evolution of high-throughput sequencing technologies, a massive number of genetic variants have been identified in numerous studies (1). Most of these identified variants are functionally neutral, and only a few lead to the occurrence of diseases (2). However, distinguishing disease-causing variants from the vast majority of passengers is still a challenge. Great efforts have been made to explore the relationship between genetic variants and human diseases. While most studies focus on nonsynonymous SNVs that alter amino acid sequences, some studies have also demonstrated that some variants function by changing the motif sequence of splice sites or influencing RNA–protein interactions by altering the secondary structure of RNA molecules (3). More recently, some studies have reported that genetic variants function by influencing RNA modifications by specifically substituting the nucleotide at the modified position or changing the nucleotide sequence in the proximal flanking region (4). Therefore, annotating genetic variants with respect to their functional consequences on RNA modifications may be a valuable strategy to decipher the pathogenic mechanisms of human diseases.

To assess the effects of variants on m^6^A, one of the most common RNA modifications in eukaryotes, in 2018, we developed m6AVar, a resource that allows the annotation, visualization and exploration of m^6^A-associated variants (5). In the previous release, m6AVar contained 1 397 244 m^6^A modification sites and 414 241 m^6^A-associated variants in humans and mice. Moreover, >2000 disease-related variants have been identified by linking the m^6^A-associated variants with GWAS and ClinVar (6) data. Since its publication, m6AVar has been widely used in studies analyzing variants by their m^6^A functions (7). Due to the importance of m^6^A modifications and the development of new high-throughput m^6^A detection methods such as m^6^A-SEAL-seq (8), m^6^A-label-seq (9), DART-seq (10) and m6ACE-seq (11), there has been explosive growth in the number of reported m^6^A sites in the last two years, resulting in a need to update m6AVar.

In addition to m^6^A, >170 different types of modifications have been reported to be present in RNA molecules until now. These kinds of modifications are involved in the regulation of various biological processes, affecting diverse activities in living cells (12). To identify these modifications in low-abundance RNA species such as mRNA, many sufficiently sensitive high-resolution transcriptome-wide techniques (e.g. Pseudo-seq, Ψ-seq, CeU-seq, RiboMeth-seq, Nm-seq, m^7^G-seq and m^1^A-MAP) have been developed (13–18). By applying these brand-new techniques, researchers have identified many novel targets of these RNA modifications, revealing their importance in regulating RNA splicing, translation, miRNA function and RNA stability (19). Furthermore, as suggested by a growing body of evidence, dysregulation of RNA modifications may be a key regulatory mechanism in a variety of diseases (20,21). It is necessary to further investigate the potential pathogenesis of all types of RNA modifications. Evaluating the effect of variants on RNA modifications will be an ideal entry point for studying the pathogenic molecular mechanisms of RNA modifications, which will further promote the identification of additional disease-causing variants.

**Figure 1.**
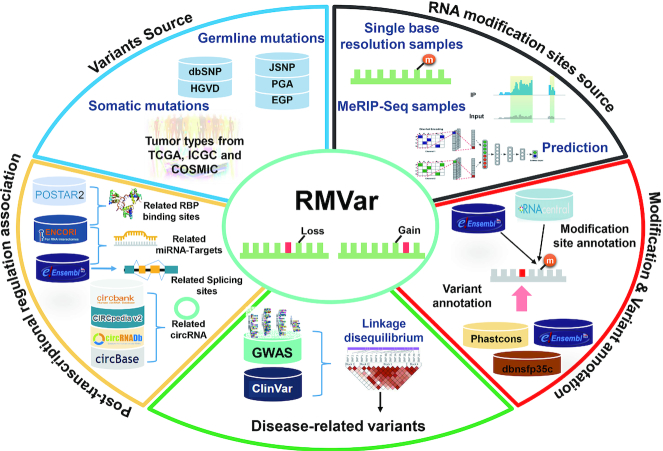
Overall design and construction of RMVar. From published literature and available databases, we collected ∼2 500 000 RNA modification sites and ∼900 000 000 genetic variants in RMVar. Using our updated procedure, the RM-associated variants were derived at three confidence levels ranging from high to low. To investigate the potential roles of RM-associated variants, annotations of posttranscriptional regulation effects were integrated. In addition, the pathogenicity of RM-associated variants was further annotated by GWAS and ClinVar data sets.

In this study, we present RMVar (http://rmvar.renlab.org) to host all RM-associated variants. An intact set of RNA modifications was collected from recently published literature. Combining the latest germline and somatic mutations with the obtained RNA modification sites, we derived RM-associated variants at three confidence levels (Figure 1). To further study the functional roles of RM-associated variants, we annotated them by determining whether they are associated with RBP binding regions, miRNA targets, splicing sites and circRNA. Finally, to explore the potential relationship between RNA modifications and diseases, the disease-associated information from the GWAS and ClinVar databases was also integrated into RMVar.

## MATERIALS AND METHODS

### Data sources

RNA modification sites were derived from both high-throughput sequencing experiments (Supplementary Table S1) and transcriptome-wide prediction based on a deep convolutional neural network model. Germline and somatic variants were collected from dbSNP (22), HGVD (23), TCGA, ICGC and COSMIC (24). To explore the underlying role of RM-associated variants in posttranscriptional regulation, the RBP binding sites from ENCORI (25) and POSTAR2 (26), the miRNA–RNA interactions from ENCORI, the canonical splice sites (GT-AG) from ENSEMBL annotations and circRNA regions from circBank (27), CIRCpedia v2 (28), circRNADb (29) and circBase (30) were integrated. In addition, we obtained a large number of disease-associated SNPs from 10 different phenotypic and -genetic data sets (ClinVar (6), NHGRI GWAS Catalog (31), Johnson and O’Donnell (32), dbGAP (33), GAD (34), denovo-db (35), Exome Sequencing Project (ESP), DisGeNet (36), GWASATLAS (37), GRASP (38)) to perform disease-association analysis. The detailed description and statistics for these data resources can be found in Supplementary Table S2.

### Data preprocessing

To unify all of the raw data under standard procedures, the genomic coordinates of all of the data resources were converted to GRCh38 or GRCm38 using the LiftOver program (39). Each modification site was annotated by multiple types of transcript structures, including CDS, 3′-UTR, 5′-UTR, start codon and stop codon. All genomic information on the noncoding genes was collected from the RNACentral database (40). In addition, we annotated all of the variants by ANNOVAR (updated 23 October 2018) in two steps as described for m6AVar.

### Derivation of RNA modification sites

To obtain RNA modification sites, we used three different strategies with confidence levels ranging from high to low, as illustrated below:

#### High confidence level

The RNA modification sites with a high confidence level were directly extracted from the published single-nucleotide resolution experiments (Supplementary Table S1). The motif (Supplementary Figure S1) for each modification type was obtained from the flanking sequence using weblogo (v3.7.4) (41).

#### Medium confidence level

The m^6^A, m^1^A and m^7^G sites with a medium confidence level were predicted from previously published m^6^A-SEAL-seq and MeRIP-seq data. First, all the m^6^A-SEAL-seq and MeRIP-seq samples from the GEO, SRA, GSA and ArrayExpress databases (Supplementary Table S1) were downloaded as raw data. FastQC (http://www.bioinformatics.babraham.ac.uk/projects/fastqc) was used to check the sequencing quality, and the sequencing adaptors were removed using Trimmomatic (v0.35) (42). A minimum of 25 nucleotides was required for unambiguous alignment. All qualified reads were realigned to reference genomes (GRCh38 for human and GRCm38 for mouse) by STAR (v2.7.0) using default parameters (43). Two peak callers, MACS2 (44) and MeTPeak (45), were applied to identify the methylation peaks separately. We used MSPC (46) to construct consensus peaks from the two methods. Single-nucleotide resolution m^6^A sites were then predicted from these consensus peaks using a deep convolutional neural network (CNN) algorithm. For m^1^A and m^7^G, single-nucleotide-resolution sites were obtained by scanning through the consensus peak regions with the corresponding modification motif using MAST (47).

#### Low confidence level

In addition, to cover all potential m^6^A sites, m^6^A sites with low confidence levels were predicted from the whole transcriptome using a CNN model (Supplementary methods).

### Derivation of RM-associated variants

We defined a variant as an RM-associated variant by evaluating whether it has the potential to alter the motifs or other sequence features essential for RNA modifications. According to various levels of confidence, we extracted the corresponding RM-associated variants using three strategies.

To derive RNA modification sites with high confidence levels, we selected the variants that were located near the high confidence level RNA modification sites and then check whether the variants destroy motifs of the RNA modification sites. Those variants that destroy the motif around the RNA modification sites with high confidence level were considered as RM-associated variants with high confidence level. Similarly, those variants that destroy the motif around the RNA modification sites with medium confidence level were considered as RM-associated variants with medium confidence level. Specifically, the MAST program from the MEME suite was used to determine whether the variant destroys the input motif.

For RM-associated sites with low confidence levels, we predicted the RNA modification status in both the reference and mutant sequences around the modification sites by our previously developed CNN model (Supplementary Methods). In general, variants resulting in the loss of RNA modification sites in the mutant sequence compared to the reference sequence were defined as RM loss-associated variants. The opposite variants were defined as RM gain-associated variants. At this confidence level, due to the lack of a prediction model for other modification types, only m^6^A-associated variants were included.

### Annotation of RM-associated variants

To study the potential role of RM-associated variants in posttranscriptional regulation, we first annotated these variants by whether they intersect with RNA-binding protein (RBP) regions, miRNA targets and splicing sites using the same strategy proposed for m6AVar. Subsequently, we matched all of the RM-associated variants with known regions of circRNAs downloaded from circBank, CIRCpedia, circRNADB and circBase to obtain potential regulatory events of RM-associated variants in circRNA formation.

To identify disease-related RM-associated variants, we annotated our resources with GWAS and ClinVar data sets following the procedure mentioned in m6AVar. For GWAS annotation, according to the previous study (48), linkage disequilibrium (LD) between genetic variants and other known disease-related mutations was computed using PLINK (49) ld-snp-list limited to windows of 1000 kb, 1 000 000 SNPs and *r*^2^ of 0.8. Only SNPs with significant association (*P* < 5 × 10^−8^) in at least one of the five superpopulations (AFR, AMR, EAS, EUR and SAS) in the 1000 Genomes Project (1KGP) were defined as GWAS disease-associated SNPs. All the RM-associated variants were then mapped with the GWAS disease-associated SNPs to annotate their disease phenotypes. Furthermore, for ClinVar annotation, we also annotated all the RM-associated variants with corresponding disease phenotypes curated in ClinVar (6).

### Database and web interface implementation

All the metadata in RMVar were stored and managed in MySQL tables. The web interfaces were implemented in Hyper Text Markup Language (HTML), Cascading Style Sheets (CSS) and Hypertext Preprocessor (PHP). To provide visualization of all the analysis results, multiple statistical diagrams were generated by EChars, and a genome browser was implemented using Jbrowser (50).

## DATABASE CONTENT

### RM-associated variants with three confidence levels

RMVar contains 1,678,126 RM-associated variants for 9 kinds of RNA modifications, namely, m^6^A, m^6^Am, m^1^A, pseudouridine, m^5^C, m^5^U, 2′-O-Me, A-to-I and m^7^G (Table 1), which is four times larger than m6Avar (Table 2). Similar to those in m6AVar, the RM-associated variants in RMVar have three confidence levels ranging from low confidence to high confidence. RM-associated variants with high confidence levels were derived from single base resolution experiments. For humans, there are 261 304 and 46 251 RM-associated germline and somatic variants with high confidence levels, respectively. For mice, there are 12 710 high confidence level RM-associated germline variants. The RM-associated variants with medium confidence levels were obtained from MeRIP-Seq experiments. At the medium confidence level, there are 518 505 human RM-associated germline variants, 110 854 human RM-associated somatic variants, and 14 079 mouse RM-associated germline variants. In addition, a genome-wide prediction based on the CNN model was performed for the sequences around all the collected germline and somatic variants to identify variants that cause potential gain or loss of m^6^A sites. As a result, we obtained 678 338 and 36 085 low confidence-level m^6^A-associated variants in humans and mice, respectively.

**Table 1.** RM-associated variants in RMVar

Confidence level^a^	Human germline variants			Mouse germline variants			Human somatic variants			Total		
	Loss^b^ variants	Gain^c^ variants	All	Loss variants	Gain variants	All	Loss variants	Gain variants	All	Loss variants	Gain variants	All
High	261 304	0	261 304	12 710	0	12 710	46 251	0	46 251	320 265	0	320 265
Medium	518 505	0	518 505	14 079	0	14 079	110 854	0	110 854	643 438	0	643 438
Low	351 982	263 233	615 215	20 236	15 849	36 085	40 063	23 060	63 123	412 281	302 142	714 423
Total	1 131 791	263 233	1 395 024	47 025	15 849	62 874	197 168	23 060	220 228	1 375 984	302 142	1 678 126

^a^Confidence levels of RM-associated variants ranging from high to low, where variants with high confidence levels were directly extracted from the single-nucleotide resolution experiments, variants with medium confidence levels were derived from MeRIP-seq experiments, and variants with low confidence levels were predicted by the CNN model.

^b^Loss variants are variants resulting in the loss of RNA modification sites in the mutant sequence compared to the reference sequence.

^c^Gain variants were conversely formed.

**Table 2.** Comparison of data resources among RMVar, m6AVar and RMBase

Features	RMVar	m6AVar	RMBase v2.0
m^6^A-associated variants	1 461 691	419 294	186 779
m^1^A-associated variants	62 492	-	1623
m^7^G-associated variants	64 867	-	7
m^5^C-associated variants	28 622	-	146
Pseudouridine-associated variants	1548	-	1243
A-to-I-associated variants	52 974	-	1
Other RNA modification type-associated variants	5932	-	2484
RBP-associated variants	655 786	219 299	165 699
miRNA target-associated variants	199 110	6 905	2758
Splicing site-associated variants	457 909	211 456	-
circRNA-associated variants	678 522	-	-
Disease-associated variants	31 076	2 637	1862
Number of Somatic RM-associated variants	220 228	62 227	192 283
Number of Germline RM-associated variants	1 457 898	352 014	-

Compared to m6AVar, in RMVar we not only included more types of RNA modification sites but also updated a more complete set of genetic variants (Table 2). For germline mutations, we integrated mutation sites from HGVD over the current dbSNP version. For somatic mutations, we not only used the latest version (updated in July 2019) of TCGA but also collected the mutation sites from ICGC and COSMIC, which is more than three times as many as the number of somatic mutations in m6AVar.

### Functional annotation of RM-associated variants

It is reported that RNA modifications sites could recruit RNA bind proteins (RBPs) to perform various molecular functions. Therefore, we further annotate the RM-associated variants by checking if they locate in the regions that have RBP binding sites, miRNA targets, splicing events and circRNAs (Supplementary Table S3).

For association analysis of RM-associated variants with RBP sites and miRNA targets, we updated the annotation information to obtain the most comprehensive association results. Compared with m6AVar, in RMVar we performed the association analysis with updated versions of StarBase (25), CLIPdb (51), ENCORI and POSTAR2 and finally obtained 1 256 212 RBP sites and 258 246 miRNA targets that were potentially altered by RM-associated variants. In this new version, there are three times more posttranscriptional annotations than those in m6AVar.

It has been reported that altering RNA modifications plays a potential role in regulating the formation of specific circRNAs in disease progression processes (52). Therefore, apart from annotating the RM-associated variants with RBP binding regions, miRNA targets and splicing events, we also integrated the potential regulatory events of RM-associated variants on circRNAs by checking whether they are located in the regions of known circRNAs. From circBank, CIRCpedia, circRNADB and circBase, we identified 678 522 RM-associated variants in 346 275 known circRNAs, providing a wealth of potential data resources to study the molecular process of circRNA formation in human diseases.

In total, for humans, 553 925 germline and 106 542 somatic RM-associated variants are related to 201 and 200 RBPs. A total of 159 427 germline and 37 144 somatic RM-associated variants are related to 194,195 and 62,888 miRNA target sites, respectively. A total of 363 375 germline and 82 651 somatic RM-associated variants are related to the splicing sites of 26 666 and 15 865 genes, respectively. A total of 561 910 germline and 108 655 somatic RM-associated variants are related to circRNAs. In mice, there are 9148 RM-associated variants related to 77 RBPs, 2539 RM-associated variants related to 4148 miRNA target sites, 11,883 RM-associated variants related to the splicing sites of 7561 genes, and 7957 RM-associated variants related to the circRNAs.

### Disease-related RM-associated variants

To link RM-associated variants to human genetic diseases, we collected disease-related variants from nine GWAS cohorts and the ClinVar database, which resulted in 5 459 281 known pathogenic variants covering 27 402 disease phenotypes. By intersecting these disease-related variants with RM-associated variants in RMVar, we found that 31 076 RM-associated variants were identified in ClinVar and GWAS (Supplementary Table S3). To link the RM-associated variants to human cancers, we intersected the RM-associated variants with the cancer somatic mutations collected in TCGA, ICGC and COSMIC, which resulted in 220 228 somatic RM-associated variants that are potentially functional in cancers.

## ENHANCED WEB INTERFACE

**Figure 2.**
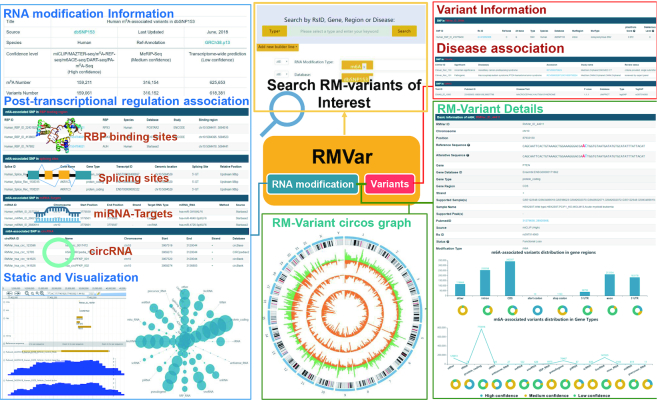
Enhanced web interface of RMVar. The search option was updated to provide more powerful extensions to the data query. The visualization of overall statistics was updated accordingly. In addition, more comprehensive information on posttranscriptional regulation related to RM-associated variants, such as RBP binding sites, splicing sites, miRNA targets, circRNAs, and RM-associated disease-related variants, is provided in the web interface of RMVar. Furthermore, the web interface presenting disease-related variants was also improved.

As presented in Figure 2, the web interface has been redesigned to be more user-friendly, faster, and esthetically pleasing in this version. RM-associated variants of interest can be directly queried by a simple search option with categories such as rsID, genes, chromosomal regions, diseases and modification types. For users who are in need of a more precise search, we have developed an advanced search option in this new version. Multiple conditions can be easily combined in the newly developed interface, and the users can obtain more specific queries compared to those in our previous version. To provide an overview of all the collected RM-associated variants in a transcriptome-wide distribution, the circos plot and a set of other statistical diagrams are presented on the Results page. Detailed information about the RNA modification sites and the associated germline and somatic variants are displayed in a redesigned interactive table, in which users can sort or query by a specific term. To further explore the functional consequences of our derived RM-associated variants, RMVar provides a brand new integrated table to present the related regulatory events, such as RBP binding, miRNA targeting, alternative splicing and circRNA formation, that may be altered by RM-associated variants. Moreover, information about the pathogenesis of each RM-associated variant is also presented in the newly developed table. Furthermore, a genome browser is implemented in RMVar to allow the presentation of transcript structures, supported reads, peaks, RNA modification sites, SNP sites and other posttranslational regulatory events for different samples. To improve readability according to user feedback, we have also rearranged our raw data stored on the download page.

## SUMMARY AND PERSPECTIVES

Currently, RMVar holds ∼1 500 000 RM-associated germline variants and ∼220 000 RM-associated somatic variants, which has expanded our data more than four times compared to m6AVar. A large fraction of the RM-associated variants were found to potentially affect RBP binding regions (39.9%), miRNA targets (11.9%), splicing sites (27.3%) and circRNA formation (40.4%). Furthermore, ∼1.9% of the RM-associated variants are identified as disease-related using a combined analysis of GWAS and ClinVar data sets.

Compared to RMBase (53), a comprehensive RNA modification database, RMVar is a specific resource for RM-associated variants and has several advantages (Table 2). First, RMBase hosts only 1862 RM-associated disease-related variants from GWAS, while RMVar contains not only 31 076 RNA modification-associated disease-related variants obtained from GWAS and ClinVar but also millions of candidate RNA modification-associated variants from germline and somatic mutation data sets. Second, RM-associated variants of RMBase are obtained from a simple position comparison with the 10 nt flanking regions of each modification site, RM-associated variants in RMVar are derived from motif alteration and deep convolutional neural network with more rigorous consideration of the effects of variants on motifs and sequence features of the modifications. Third, RMVar contains more comprehensive functional annotation of RM-associated variants that includes RBP binding sites, miRNA targets, splicing events and circRNAs, while RMBase only annotates the relationships between RM-associated variants, RBPs and miRNA binding sites.

As a long-term goal, we would like to continually update RMVar and increase important RM-associated variant information to cover as many types of RNA modifications as possible. In the future, as new high-throughput epitranscriptome sequencing data sets are constantly added to RMVar, we would like to develop an automatic pipeline in the curation and annotation process to increase our capacity to integrate the information of these data sets. Continuous improvements will also be made in the database server performance for storing and visualizing these new incoming data. We will maintain RMVar to ensure that it remains a useful resource for the research community.

## DATA AVAILABILITY

RMVar is a comprehensive online database available at http://rmvar.renlab.org.

## Supplementary Material

gkaa811_Supplemental_Files
