# Supplementary material for: RMVar: an updated database of functional variants involved in RNA modifications
Source: Nucleic Acids Res. 2020 Oct 6;49(D1):D1405–12. doi: 10.1093/nar/gkaa811 (PMC7779057; doi:10.1093/nar/gkaa811)
Supplement: gkaa811_Supplemental_Files [file gkaa811_supplemental_files.zip › Supplymentary data.pdf]

RMVar: an updated database of functional variants involved in RNA modifications

Xiaotong Luo[1,#], Huiqin Li[1,#], Jiaqi Liang[1], Qi Zhao[1], Yubin Xie[2,*],Jian Ren[1,*], Zhixiang Zuo[1,*]

[1] State Key Laboratory of Oncology in South China, Cancer Center, Collaborative Innovation Center for Cancer Medicine, School of Life Sciences, Sun Yat-sen University, Guangzhou 510060, China
[2] Precision Medicine Institute, The First Affiliated Hospital, Sun Yat-sen University, Guangzhou 510060, China

[*]Correspondence to: Zhixiang Zuo (zuozhx@sysucc.org.cn); Jian Ren (renjian.sysu@gmail.com); Yubin Xie (xieyb6@mail.sysu.edu.cn).

[#] The authors wish it to be known that, in their opinion, the first two authors should be regarded as joint First Authors.

SUPPLEMENTARY METHODS

Single-nucleotide resolution prediction of m^{6}A sites based on the convolutional neural network algorithm

We developed a convolutional neural network model for identification of m^{6}A sites at single-nucleotide resolution level. The development and performance of the model are characterized as follows.

Data collection: To construct models for predicting precise m^{6}A sites by primary sequence, we first obtained the single-base-resolution m^{6}A sites from two recently published miCLIP experiments. Following the same processing approach in our previously published work (1), we constructed a nonredundant training set with 245,023 m^{6}A sites for the human model and another 181,690 m^{6}A sites for the mouse model (2).

All of the above collected m^{6}A sites were regarded as a positive training set. The negative datasets were generated according to the positive sets. Using the positive datasets as references, we extracted the nonmethylated adenines that conformed to a consensus DRACH motif (where D=A, G or U; R=G or A; H=A, C or U) in the same exon as the negative dataset (2,3). As presented in other related studies, the m^{6}A sites were not randomly distributed in a methylated transcript; instead, they were prominently enriched around stop codons. To consider this point in our study, we further filtered the extracted negative sites by preserving those located within 200 bp near known m^{6}A sites. Finally, a total of 246,491 and 257,404 non-m^{6}A sites were constructed as the negative training set for both the human and mouse models, respectively.

Construction of the m^{6}A site prediction models: Before the training step, a critical process is transforming the collected modification sites into a set of feature matrices that can be directly recognized by neural network algorithms. As presented in our previously published papers (4), the primary sequence composition around the modified residue may affect the specificity of biomacromolecular modification. Therefore, to capture the sequence dependency of m^{6}A

events, we first constructed the encoding scheme by extracting features from the flanking sequence of length 65 bp in which the DRACH motif is located at the central position.

Next we encoded each site into a 4×65 dimensional matrix using the one-hot encoding scheme. To further capture the sequence conservation of the m^6A process, we applied our previously proposed PSSM encoding method (4) to calculate the tendency score between m^6A sites and non-m^6A sites. According to the computational pipeline, we constructed an encoding matrix that can represent the nucleotide preferences at each position. If a given score in the encoding matrix is greater than zero, then the nucleotide at this position is more likely to be observed in the positive set; conversely, the nucleotide would have a better chance of appearing in the negative set. Using this encoding matrix, we filled the one-hot matrix with its corresponding conservation score and constructed a one-channel feature matrix for training and predicting.

To perform m^6A site prediction, a CNN model was adopted in our prediction algorithm. The CNN model consisted of 7 neural network layers. The first layer is a convolutional layer with dimensionality in 4×65. For the primary sequence model, the convolutional layer accepts a feature matrix with a single channel as input data. The second layer is the pooling layer. Here, we used a max-pooling method to downsample an input representation with reduced dimensionality. Following the max-pooling layer, we implemented another four fully connected layers to further compress the extracted features. To avoid gradient diffusion during the training process, the ReLU function was adopted as the activation function in each fully connected layer. A dropout approach was also introduced in the fully connected layer for overfitting reduction. To further classify the extracted features, the last layer of our CNN model was implemented as a softmax layer. We used a softmax classifier to compute the probability of m^6A sites and non-m^6A sites.

Before using the CNN model for prediction, we first had to optimize its parameters based on the training dataset. To achieve this goal, the negative log-likelihood was considered as a loss function in our optimization step. The L1 and L2 regularization were also integrated to enhance robustness. We then applied the mini-batch stochastic gradient descent algorithm with a batch

size of 80 to update network parameters during the back-propagating process. The aforementioned network was implemented and trained by the deeplearning4j library in JAVA.

Prediction of m^6A-associated variants in low confidence level: By integrating all of the exonic variants into the m^6A site prediction model, we predicted the m^6A status of the same sample in the reference sequence and mutant sequence. The m^6As that occurred in the reference sequence and lost in the mutant sequence were defined as m^6A-loss alterations. In the opposite case, they were defined as m^6A-gain alterations.

SUPPLEMENTARY REFERENCES

1. Jiang, S., Xie, Y., He, Z., Zhang, Y., Zhao, Y., Chen, L., Zheng, Y., Miao, Y., Zuo, Z. and Ren, J. (2018) m6ASNP: a tool for annotating genetic variants by m6A function. *Gigascience*, **7**.
2. Ke, S., Alemu, E.A., Mertens, C., Gantman, E.C., Fak, J.J., Mele, A., Haripal, B., Zucker-Scharff, I., Moore, M.J., Park, C.Y. *et al.* (2015) A majority of m6A residues are in the last exons, allowing the potential for 3' UTR regulation. *Genes Dev*, **29**, 2037-2053.
3. Meyer, K.D., Saletore, Y., Zumbo, P., Elemento, O., Mason, C.E. and Jaffrey, S.R. (2012) Comprehensive analysis of mRNA methylation reveals enrichment in 3' UTRs and near stop codons. *Cell*, **149**, 1635-1646.
4. Xie, Y., Luo, X., Li, Y., Chen, L., Ma, W., Huang, J., Cui, J., Zhao, Y., Xue, Y., Zuo, Z. *et al.* (2018) DeepNitro: Prediction of Protein Nitration and Nitrosylation Sites by Deep Learning. *Genomics Proteomics Bioinformatics*, **16**, 294-306.

SUPPLEMENTARY FIGURES

Figure S1 – Motifs of 9 major types of RNA modifications discovered by WebLogo. The motif region for each modification type were derived from the flanking sequence of RNA modification sites having a high confidence level.

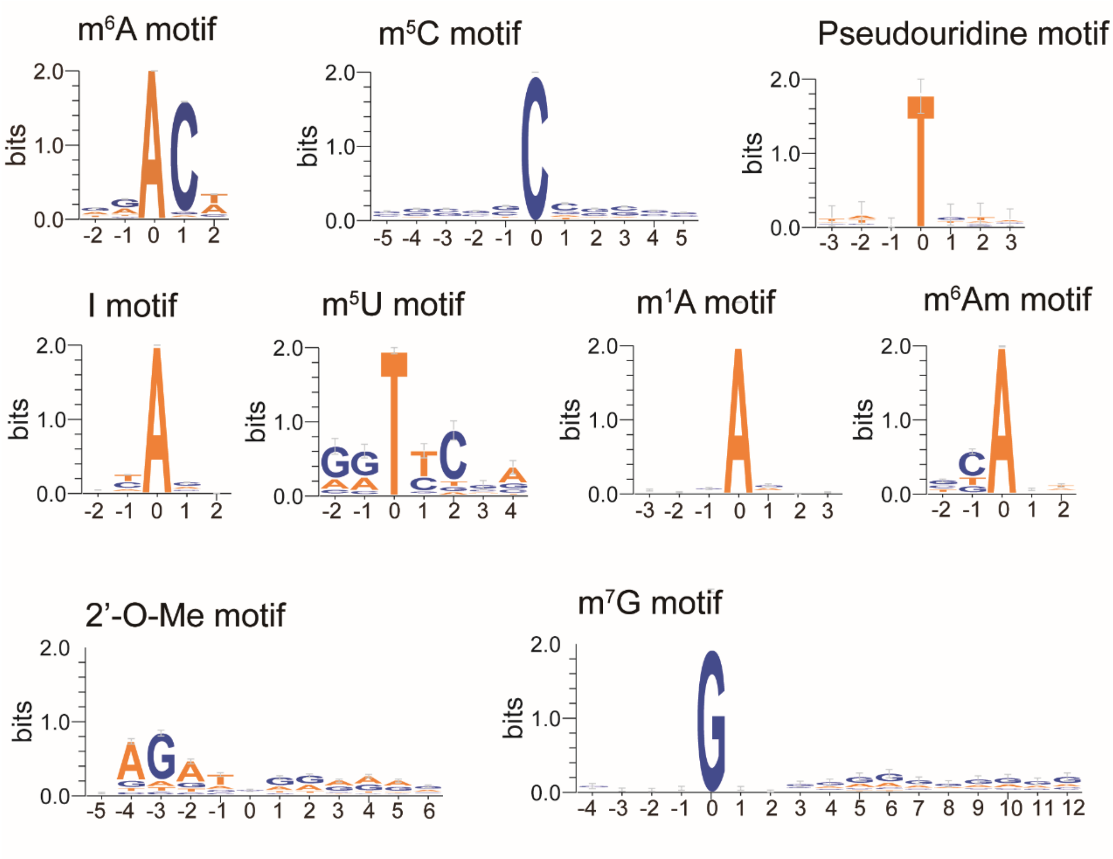

SUPPLEMENTARY TABLES

Table S1 – High throughput sequencing data sets collected in RMVar

Table S2 – Overview of data resources in RMVar

Table S3 - Statistics of associated data resources in RMVar
